# Mindfulness training reduces the preference for proenvironmental outcomes

**DOI:** 10.1038/s41598-024-79137-0

**Published:** 2024-11-27

**Authors:** Zarah Le Houcq Corbi, Kathrin Koch, Britta Hölzel, Alexander Soutschek

**Affiliations:** 1 Department of Psychology, Ludwig-Maximilians-Universität München, Leopoldstr. 13, 80802 Munich, Germany; 2 Department of Neuroradiology, Technical University of Munich, Munich, Germany

**Keywords:** Proenvironmental behavior, Mindfulness, Delay discounting, Social discounting, Drift diffusion model, Human behaviour, Environmental social sciences

## Abstract

Theoretical accounts posit that mindfulness promotes proenvironmental behavior. While this claim is supported by correlational findings, past intervention studies provided no evidence that enhancing mindfulness increases self-report measures of proenvironmental behavior. Here, we tested whether a 31-day mindfulness intervention strengthens preferences for proenvironmental outcomes with decision tasks involving real conflict between participants’ selfish interests and beneficial consequences for the environment. To unravel the psychological mechanisms underlying the impact of mindfulness on proenvironmental behavior, we assessed the impact of mindfulness training on prosociality and future orientation. Contrary to our hypotheses, the mindfulness intervention reduced instead of increased preferences for proenvironmental and prosocial outcomes, whereas no effects were observed on future orientation. Baseline preferences for proenvironmental and prosocial outcomes (and the intervention effects on them) were correlated, providing empirical evidence for a link between proenvironmental behavior and prosociality. Together, the current data suggest that the relationship between mindfulness and proenvironmental behavior as well as prosociality may be more complicated than assumed in the literature.

**Supplementary Information:**

The online version contains supplementary material available at 10.1038/s41598-024-79137-0.

## Introduction

The global climate crisis is characterized by unprecedented annual increases in greenhouse gas (GHG) emissions, particularly carbon dioxide, with significant implications for the environment and biodiversity^1^. Individual behavior plays a crucial role in driving these emissions^2,3^: in Germany, for example, an average of eight to nine tons of CO_2_ is emitted per person and year (German Federal Ministry for the Environment, Nature Conservation, Nuclear Safety and Consumer Protection (BMUV); Federal Statistical Office of Germany), underlining the need for individuals to make more proenvironmental choices to reduce their carbon footprint^4^. It is therefore important to obtain a better understanding of the psychological determinants of proenvironmental behavior^5,6^. Theoretical accounts suggest a link between proenvironmental behavior and mindfulness^7^. The current study therefore tested the hypothesis that mindfulness practice strengthens the preference for proenvironmental behavior.

Mindfulness is a multifaceted concept: on the one hand, it refers to a psychological disposition, often termed dispositional mindfulness, which represents an individual’s inherent ability to maintain attention on the present moment without judgment^8^. On the other hand, mindfulness can also be cultivated through specific practices designed to enhance this ability, referred to as mindfulness interventions^9^. While dispositional mindfulness and mindfulness interventions are related, they are also distinct. Dispositional mindfulness represents a stable trait that differs among individuals, whereas mindfulness training involves active engagement in practices aimed at developing or strengthening this trait. The eight-week Mindfulness-Based Stress Reduction (MBSR) program, created by Kabat-Zinn, is the most widely used mindfulness-based intervention in research and has been shown to increase dispositional mindfulness^10,11^. Mindfulness-based interventions can modulate different mental states depending on the specific training used, including attentional control, non-reactivity, present-moment awareness, acceptance, non-judgment, and compassion^12^.

Proenvironmental behavior refers to behaviors intended to protect or avoid harm to the environment^6^. Research has implemented various methods to measure proenvironmental behavior, with self-reported measurements being the most common^13^. However, these methods are often criticized for their limitations, including response biases, inconsistency, and social desirability effects^14^. Furthermore, they tend to focus on environmental attitudes—often referred to as moral concern for the environment—rather than actual environmental impact^15,16^. Given the attitude-behavior gap, where proenvironmental attitudes rarely translate into corresponding behaviors, it is crucial to differentiate between proenvironmental attitudes and behaviors^4^. An alternative approach is to assess proenvironmental behavior using experimental paradigms. These paradigms allow for the investigation of environmental consequences directly resulting from individuals’ proenvironmental behavior and facilitate the testing of interventions in a controlled setting before applying them to real-world practices^16^.

Mindfulness has been hypothesized to promote proenvironmental behavior^7^. Theories posit that mindfulness could disrupt unsustainable habits by reducing automatic responses and emotional reactivity as well as by enhancing the congruence between values and behaviors, thereby fostering proenvironmental behavior^17–22^. However, the hypothesized mindfulness-environmental link is only partially supported by empirical evidence. A recent meta-analysis reported only a small positive correlation between dispositional mindfulness and proenvironmental attitudes^23^. Similarly, another study found a link between dispositional mindfulness and self-reported proenvironmental behavior^24^. While these correlational results indicate an association between individual differences in mindfulness and self-reported environmentally friendly behaviors, they cannot determine whether mindfulness causally modulates proenvironmental behavior. To address this, Tang et al. (2017) conducted an intervention study comparing the effects of mindful learning to mindless learning on proenvironmental behavior and found that mindful learning led to higher self-reported proenvironmental attitudes compared to the control training^25^. Another study using a similar intervention reported that participants in the mindful learning condition exhibited stronger beliefs in climate change compared to those in the mindless learning condition^26^. While these results underscore the importance of mindfulness in promoting proenvironmental attitudes, they do not reliably determine whether mindfulness can reduce human impact on climate change. This is because proenvironmental attitudes do not consistently predict corresponding behaviors or their environmental impact^4,27^. A recent study, for instance, revealed that the relationship between attitudes and behavior is often influenced by factors such as perceived personal cost and environmental benefit^28^. Previous intervention studies, however, provided mixed evidence for an influence of mindfulness training on proenvironmental behavior. One intervention study implementing an eight-week mindfulness program observed no direct effects on self-reported sustainable consumer behavior^29^. In contrast, a pilot study found that an eight-week program combining elements of environmental education and mindfulness reduced participants’ estimated carbon footprints, based on self-reported behaviors^30^. However, due to the small sample size (i.e., 16 participants), these results should be considered as preliminary^30^. Furthermore, when using a similar carbon footprint estimation method, an eight-week mindfulness program without educational components showed no direct effects on proenvironmental behavior in naïve meditators^31^. In contrast to correlational studies which suggest a positive relationship between mindfulness and proenvironmental attitudes, evidence for a direct effect of mindfulness-based interventions on proenvironmental behavior is lacking. To address this gap, the current study causally tested the direct effects of a mindfulness intervention on proenvironmental behavior using experimental tasks that involve real-world environmental consequences such as carbon emissions and financial costs.

A further goal of our study was to unravel the psychological mechanisms underlying a potential influence of mindfulness on proenvironmental behavior. In particular, we focused on prosociality and future-orientation as possible mediators of the mindfulness-environment relationship. Theoretical accounts posit close links between concerns for environment and society^32^, because proenvironmental actions require individuals to weigh their selfish interests against the preservation of natural resources; the latter incurs no direct benefits for the individual but mainly for others, in particular future generations. In line with this assumption, proenvironmental behavior is correlated with compassion, which is thought to be related also to prosocial behavior^33–35^. Given the evidence that mindfulness training enhances empathy and compassion^36,37^, and that their neural substrates have been shown to be causally linked to proenvironmental^38^ and prosocial behavior^39–41^, mindfulness training may therefore influence also prosociality and proenvironmental behavior mediated via its effects on compassion. Furthermore, meta-analytical evidence suggests that mindfulness interventions promote prosociality^42^. It seems therefore plausible to assume that mindfulness may promote proenvironmental behavior via enhancing prosociality.

Another cognitive mechanism that might connect mindfulness to proenvironmental behavior is future-orientation. Previous research suggests that mindfulness might promote future-oriented behavior, although this effect may be stronger for primary than for secondary rewards^43^ and may depend on individual differences in baseline impulsiveness^44^. Future-oriented behavior is also conceptually linked to proenvironmental behavior because it requires resisting immediate temptations (like indulging in a long shower or going by car) in order to achieve long-term proenvironmental goals benefitting future generations^45^.

To investigate whether mindfulness-based training promotes proenvironmental behavior via strengthening prosociality and/or future-orientation, we conducted a pre-registered study where mindfulness-naïve participants performed decision tasks measuring proenvironmental, prosocial, and future-oriented preferences before and after a mindfulness or control training (pre-test/post-test design). We hypothesized mindfulness (relative to active control) training to increase preferences for proenvironmental outcomes (hypothesis 1). Moreover, as potential mediators of the influence of mindfulness on proenvironmental behavior, we expected the mindfulness training to strengthen also prosociality in social decision making (hypothesis 2) and explored potential intervention effects on future orientation in an intertemporal decision task.

We measured proenvironmental preferences with two experimental tasks: an environmental decision task and an environmental donation task. In the environmental decision task, participants made choices between a proenvironmental (reducing carbon emission) and a monetary option (monetary bonus for the participant with less or no reduction of carbon emission). In the environmental donation task, participants could donate money to different environmental organizations. Contrary to previous mindfulness studies assessing proenvironmental behavior with self-report questionnaires^23^, these tasks allowed testing the impact of mindfulness on decisions involving real-world implications for one’s own benefits and environmental consequences.

## Materials and methods

### Participants

Eighty-six participants were recruited through the participant pool of the Munich Experimental Laboratory for Economic and Social Sciences at the Ludwig Maximillian University of Munich. During the recruitment, participants were informed that we would be testing the influence of health awareness on decision-making and avoided mentioning “mindfulness” or “meditation” in the study description. Four participants were not invited to the post-test session because of incomplete training performance (> 4 missed training sessions). Therefore, the final sample included eighty-two participants ranging from 18 to 35 years (mean age = 23.3, ranging from 18 to 35 years, 63 women, 19 men), with 40 in the control group (mean age = 23.4 years, 31 women, 9 men) and 42 in the mindfulness group (mean age = 23.2 years, 32 women, 10 men). Our sample is highly educated, with 67% holding a university entrance diploma, 32% having completed university, and 1% possessing a vocational school diploma. We excluded participants with prior history of psychiatric or neurological diseases and with prior mindfulness experience in the last three years as recent experiences with mindfulness might potentially reduce the effectiveness of our training. Prior to participation, participants gave written informed consent. The study was approved by the ethics committee of the Ludwig Maximillian University of Munich (31_Soutschek_b), performed in accordance with the Declaration of Helsinki, and preregistered on OSF (https://osf.io/3wbs4).

### Study design and procedures

**Fig. 1:**
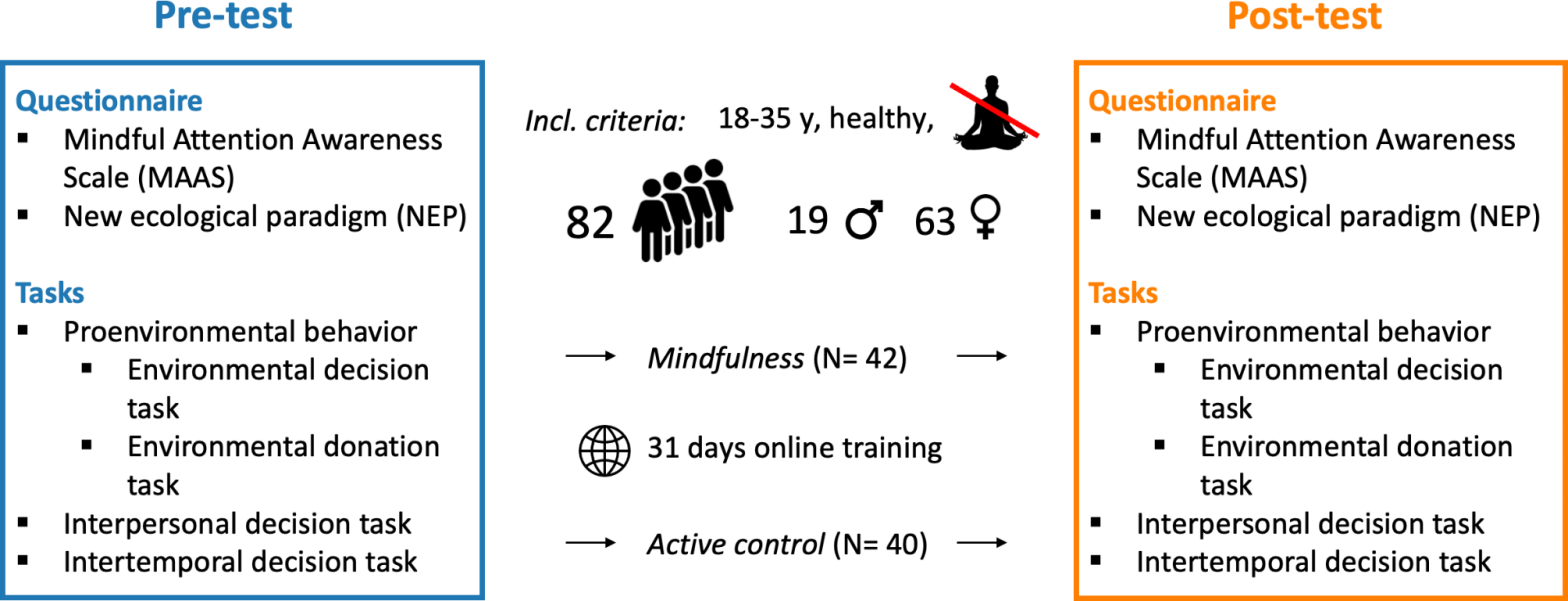
Experimental Design. The experimental group completed a 31-day online mindfulness training, while the active control group undertook a health enhancement training. Before and after the training, participants completed questionnaires assessing their mindful attention awareness state (MAAS) and environmental attitudes (NEP). Additionally, they completed two tasks: an environmental decision task and an environmental donation task as measures of proenvironmental behavior as well as an interpersonal decision task and an intertemporal decision task as measures of prosociality and future orientation, respectively.

The study followed a pre-test/post-test design where participants were pseudorandomly assigned to either the mindfulness or the control group. Before the start of the recruitment, we randomly assigned testing slots to either the mindfulness training group or the control training group. Participants registered for these slots without knowing which group they would be in and received the training corresponding to their assigned slot. An unbiased team member managed the session assignments, ensuring that the researchers remained unaware of which participants were in which group. During recruitment, participants were informed that they would be participating in a study related to health topics. Both groups conducted an online training, the first group completed a mindfulness training and the latter an active control training that focused on health enhancement. Both trainings were thirty-one days long and included daily fifteen-minute sessions that could be completed on a smartphone or computer. The daytime and location of the training were chosen by the participants. We tracked participants’ training completion using an online platform, monitoring their adherence to the specified timeframe for training sessions (i.e., fifteen minutes per day). Before (pre-test) and after (post-test) the training, participants completed computer-based tasks in the lab measuring proenvironmental, prosocial, and future-oriented preferences as well as questionnaires assessing participants’ mindful state and proenvironmental attitudes (Fig. 1). All computer-based tasks and questionnaires were presented in counterbalanced order to minimize the risk of confounding training with task order effects.

#### Mindfulness training

 We used an adapted version of an online training (in German) that had been implemented in past studies^46,47^. The training involved daily active exercises presented in various formats such as videos, audio, or texts. During thirty-one days, different meditation techniques were repeated: (1) traditional methods such as breathing meditation, body scan, and body sensation, in total nine sessions, (2) meditations targeting emotions (e.g., loving-kindness), in total four sessions and (3) sessions focused on metacognitive aspects such as open awareness and seating in silence, in total six sessions. The remaining days included videos describing psychological processes applied during mindfulness like being in the present moment, communicating mindfully, or contemplating who you are. While the original version of the training largely focused on attention to body sensations, we modified it to strengthen the balance between different kinds of meditations. In particular, we changed the order of the meditations to have diversity throughout the training, erased the walking and hearing meditation, and added instead more meditation sessions focussing on metacognitive aspects, such as awareness. The mindfulness training was structured to gradually introduce participants to a variety of mindfulness techniques and concepts over 31 days. The program started with an introduction to mindfulness on day 1, followed by breathing exercises on days 2, 3, 5, and 6. Day 4 provided guided practices on arriving in the present moment, while days 7 and 8 focused on exercises targeting the awareness of thoughts (revisited on days 22 and 27). Days 9, 10, and 12 introduced body scan techniques, and day 11 explored the relativity of perception. Days 13 and 14 centered on body sensations, and day 15 introduced the role of evaluation in mindfulness. Open awareness practices were introduced on days 16 and 17 (repeated on day 29), followed by an introduction to mindful communication on day 18. Days 19 and 20 addressed managing feelings mindfully, and day 21 explored the concept of turning towards challenges instead of away from them. Next, sessions on silent sitting were held on days 23 (revisited on day 30 as well), and days 24, 25, and 26 focused on cultivating loving kindness. Lastly, day 28 encouraged participants to reflect on the question “Who Am I?“, and day 31 concluded the program with final reflections and closing remarks.

#### Control training

The active control training (adopted from Bremer et al.^46^) included health enhancement topics such as sleep hygiene, stress management, or dietary advice and contained informative videos, audio, and texts extracted from popular science broadcasting formats.

### Behavioral assessments

#### Environmental decision task

**Fig. 2:**
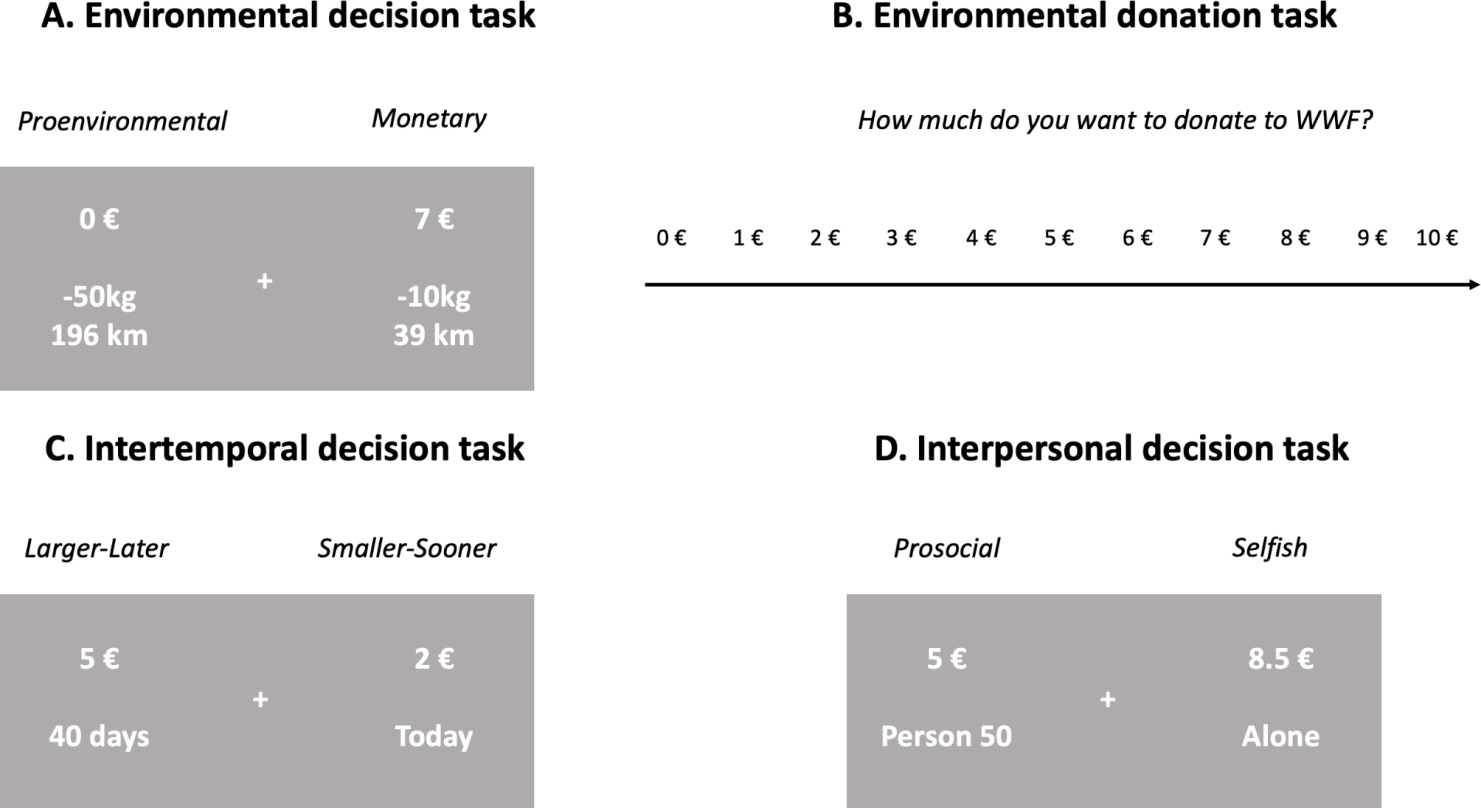
Illustration of decision tasks. Two tasks measured proenvironmental behavior: (**A**) In the environmental decision task, participants chose between a proenvironmental option (no reward but higher carbon dioxide reduction) and a monetary option (reward with a small or no carbon dioxide reduction). (**B**) In the environmental donation task, participants had to indicate how much money they would like to donate to a specific environmental charity, for example, the WWF, on a rating scale. Moreover, (**C**) the intertemporal decision task required choices between larger-later and smaller-sooner rewards, whereas (**D**) the interpersonal decision task required choices between a prosocial option (sharing money with another person) and a selfish option (keeping money for oneself).

In the environmental decision task (adapted from Berger and Wyss, 2021^48^), participants chose between options with different consequences for their monetary payoff and the environment. The proenvironmental option was not associated with a monetary reward for the participants but with a reduction of a certain amount of carbon dioxide emission (ranging from − 0.1 to -50 kg). In contrast, the monetary option included a monetary reward for the participant (1 to 10 €) but a lower reduction of carbon dioxide emission (ranging from 0 to -10 kg) than the proenvironmental option (Fig. 2A). Thus, in this task participants were confronted with a conflict between their selfish payoff and beneficial consequences for the environment. The choice options were randomly presented on the left or right screen side and participants selected their preferred option by pressing the corresponding arrow key (left or right). It is important to emphasize that the chosen options had real consequences for participants’ payoff and the environment. Participants were informed before the start of the tasks that one of the trials would be randomly selected at the end of the experiment and the chosen amount of money was added to participants’ payment; this procedure applied to all the tasks of the experiment. For choices that included a carbon dioxide reduction, we bought CO_2_ certificates of the displayed amount and destroyed them to take them out of the market, thereby removing them from the European Emissions Trading System and reducing real-life CO_2_ emissions. We informed participants about this procedure before the task and emphasized they should take every choice seriously because all trials were equally likely to be selected after the experiment. We moreover informed participants that carbon dioxide is an important contributor to climate change and we translated the amount of reduced carbon dioxide emissions into the number of kilometers an average car has to drive to emit the given amount of carbon dioxide to help participants to better understand the real-world consequences of their choices. In addition, participants were given some references to help them understand the magnitude of the kg amounts. For example, they were informed that an average German emits between nine to ten tons of CO_2_ per year or that a flight from Munich to Rome emits around 140 kg of CO_2_.

#### Environmental donation task

As a further measure of proenvironmental preferences, participants also performed a donation task where they could donate an amount of money between 0 and 10 € to an environmental organization. Participants were informed that they could receive €10 from us and that one trial could be randomly selected at the end of the experiment. The chosen amount of money would be donated, while the remaining non-donated portion of the 10 € endowment would be added to their final payoff. The amount participants were willing to donate had to be indicated on an 11-point rating scale from 0 to 10 € (Fig. 2B). The task included a total of four trials with four different environmental organizations. Before the task participants received an overview of the goals and values of each organization. The following four organizations were included: WWF (World Wide Fund For Nature), NABU (Naturschutzbund Deutschland), BUND (Bund für Umwelt und Naturschutz Deutschland), and Primaklima.

#### Intertemporal decision task

As a measure of future-oriented preferences, we administered an intertemporal choice task which required participants to make choices between smaller immediate and larger later monetary rewards. The smaller immediate rewards ranged from 0.5 to 4.5 € in steps of 0.5 (nine immediate reward levels), whereas the larger later reward was fixed to 5 €, with the temporal delay varying from 2 to 360 days (six delay levels) (Fig. 2C). The choice options were randomly presented on the left or right screen side and participants selected the preferred option by pressing the left or right arrow key for the option presented on the left or right screen side, respectively.

#### Interpersonal decision task

In this task^49,50^, we first asked participants to imagine a scale ranging from 1 to 100 representing the closeness of their relationships to other individuals in their lives (i.e., social distance). The number ”0” referred to themselves, “1” to someone very close to them (e.g., their mother), “50” to someone they have seen repeatedly, but do not know their name, and “100” to a stranger on the street. Participants were asked to avoid thinking about relationships that caused negative feelings. During the task, participants then decided between a selfish option involving an amount of money for themselves only and a prosocial option where they shared the money with another individual at varying social distances. The reward of the selfish option ranged from 5 to 10 € in steps of 0.5. In the case of the prosocial option, both the participant and the other person received 5 €, and we used the social distances of 1, 5, 10, 20, 50, and 100 (Fig. 2D). Again, the decisions were indicated with the right and left arrow keys, and the screen presentation sides of both options were counterbalanced.

#### Control measures

To measure the effectiveness of the mindfulness training, we used the Mindful Attention Awareness Scale (MAAS)^51,52^, which is a validated self-report questionnaire for participants’ mindfulness state (i.e., their ability to stay present in their daily life experiences). Moreover, participants also completed the Positive and Negative Affect Schedule (PANAS) test^53^ to control for training effects on mood. We also used a computer-based version of the digit span backward task as a measure of working memory capacity. Lastly, we controlled for side effects of mindfulness by using a German version of the Meditation-Related Adverse Effects Scale^54,55^. Lastly, to measure participants’ proenvironmental attitudes, we implemented the New Ecological Paradigm (NEP) questionnaire^56^, which consists of 15 statements about the relationship between humans and the environment. We used the NEP sum score for statistical analyses.

### Statistical analyses

Statistical analyses were conducted with R version 4.3.2. We analyzed data in the decision tasks both with model-free and model-based (drift diffusion modelling (DDM)) analyses. For the model-free analyses, we conducted generalized linear mixed models (GLMMs) that regressed binary choices in the proenvironmental, intertemporal, and interpersonal decision tasks on fixed-effect predictors for Group (0 = control, 1 = mindfulness), Session (0 = pre, 1 = post) and the interaction using the function glmer in the lme4 package. Session was also modeled as random slope in addition to participant-specific random intercepts. Similarly, we also analyzed log-transformed reaction times in these tasks with the lmer function and the additional predictor Choice as well as all interaction effects.

In addition, for both the interpersonal and intertemporal choice tasks, we calculated hyperbolic discount functions which indicate how the subjective values of shared and delayed rewards decline with increasing social distance or temporal delay, respectively. We used the hBayesDM package in R to estimate hyperbolic discount parameters separately for the pre-test and post-test data, assuming a standard hyperbolic discount function:

$$\:SV=\frac{reward\:magnitude}{1+k\times\:social\:distance\:/temporal\:delay}$$

where SV is the discounted subjective value of the shared or delayed reward and k is an individual-specific constant that quantifies the degree of hyperbolic discounting (“discount factor”). We converted subjective values into binary choices using a softmax function with the inverse temperature parameter β_temp_:

$$\:P\left(choice\:of\:shared\:or\:delayed\:reward\right)=\frac{1}{1+\text{e}\text{x}\text{p}(-{\beta\:}_{temp}\times\:(SV-selfish\:/immediate\:reward\left)\right)}$$

We estimated the parameters k and β_temp_ in a hierarchical Bayesian fashion (2 chains with 4,000 samples, the first 1000 samples were used as burn-in) and log-transformed the resulting individual parameter estimates for the statistical analysis.

For the environmental donation task, the digit span task, as well as the MAAS and NEP questionnaires, we conducted linear regressions (function lmer) where the dependent variable was predicted by fixed-effect predictors for Group, Session, and the interaction term in addition to participant-specific random intercepts. We note that all findings based on general linear regressions were robust to using non-parametric rather than parametric regression models. For the meditation-related adverse effects, we used a chi-square test for binary data (adverse effect present vs. absent) to determine whether adverse effects occurred more often in the mindfulness than in the control group.

In addition to the model-free analyses, we analyzed data in the decision tasks with exploratory (not pre-registered) hierarchical Bayesian drift-diffusion models (DDMs). The DDM, unlike the generalized linear model (GLM), simultaneously accounts for both decision choices and reaction times, providing a more nuanced understanding of the subcomponents of the decision-making process. DDMs simulate decisions as an evidence accumulation process that starts after a non-decision time (τ) and continues until a decision boundary (α) is reached. In our analysis, the lower and upper decision boundaries were associated with monetary and proenvironmental choices, respectively, in the environment decision task, with selfish versus prosocial choices in the interpersonal decision task, and with choices of immediate versus delayed rewards in the intertemporal decision task. The speed of this evidence accumulation process, known as the drift rate (ν), depends on the preference strength for one option over the other. By incorporating these parameters, DDMs offer a comprehensive view of the underlying cognitive mechanisms driving decision-making and allow determining which subcomponent of the decision process was affected by our mindfulness training. For the DDM analysis, we used the JAGS software package^57^, which utilizes Markov Chain Monte Carlo sampling to estimate the DDM parameters ν (drift rate), α (decision boundary), ζ (starting bias), and τ (non-decision time)^58^. Following previous procedures^59^, we assumed that the speed of the drift rate (ν) is given by a linear combination of the individually weighted influences of reward magnitudes and action costs (i.e., carbon dioxide emission, social distance of recipient, or delay of reward delivery). We also modeled how the mindfulness and control interventions changed the accumulation process in the post-test relative to the pre-test. For example, in the environment decision task the drift rate ν was given by the following equation:

$$\upnu = {\upbeta _1}\left( {{\text{Reward}}} \right) + {\upbeta _2}\left( {{\text{Session}} \times {\text{Reward}}} \right) + {\upbeta _1}\left( {{\text{C}}{{\text{O}}_{2{\text{diff}}}}} \right) + {\upbeta _1}\left( {{\text{Session}} \times {\text{C}}{{\text{O}}_{2{\text{diff}}}}} \right)$$

Here, Reward is the monetary reward associated with the monetary option, CO_2diff_ is the difference in CO_2_ emission reduction between the proenvironmental and the monetary option. For the interpersonal and intertemporal decision tasks, we modified Eq. 3 by replacing Reward and CO_2diff_ with the differences in reward magnitudes and social distances/temporal delays between the choice options.

We modeled training effects also on all other DDM parameters. For example, the training effect on the starting bias parameter (and analogously for the decision boundary and non-decision time) was given by:

$$\upzeta = {\upbeta _5} + {\upbeta _6}\left( {{\text{Session}}} \right)$$

To investigate group differences between DDM parameters, we modeled both individual and group-level parameters separately for the mindfulness and the control group in a hierarchical Bayesian fashion. Individual parameters were assumed to be normally distributed around group-level parameters. To test for significant group differences, we computed the differences between the posterior parameter distributions of the group-level parameters for the session effects (which capture the difference between post-test and pre-test parameter estimates) in the mindfulness and the control group. If the 95% highest density interval (HDI_95%_) of this difference did not entail zero, the group difference was considered statistically significant. We excluded trials with unreasonable fast decision times below 250 ms^60^. As priors, we assumed non-informative uniform priors over plausible parameter ranges and estimated parameters by computing two chains with 20,000 samples (burning = 15,000). $\:\widehat{R}$ was below 1.01 for all parameter estimates, indicating model convergence.

## Results

### Mindfulness training increased participants’ mindful state

As a sanity check, we first assessed whether the mindfulness training enhanced participants’ mindful state compared to the control training as measured with the MAAS. The mindfulness training increased mindfulness scores compared to the control training, β = 0.29, *t*(80) = 2.31, *p* = 0.024. A post-hoc analysis revealed an increase in mindfulness scores in the mindfulness group from the pre-test to the post-test, β = 0.30, *t*(41) = 2.80, *p* < 0.01, whereas the control group showed no significant change, β = 0.01, *t*(39) = 0.16, *p* = 0.87. In contrast, we found no significant training effects on positive, β = -0.22, *t*(80) = 1.43, *p* = 0.16, or negative mood (measured with the PANAS), β = -0.18, *t*(80) = 1.12, *p* = 0.27, or on working memory capacity, β = 0.11, *t*(80) = 0.34, *p* = 0.74. The treatment groups also showed no significant differences in age, *t*(78) = 0.31, *p* = 0.76, or gender, *t*(80) = 0.14, *p* = 0.89, and participants in the mindfulness group did not report more adverse effects (MRAEs questionnaire) than the control group, chi-square test: *χ*^2^(1) = 2.35, *p* = 0.13. Thus, any potential training effects on the decision tasks cannot be explained by such confounding variables but are likely to result from the increased mindfulness in the mindfulness compared with the control group.

### Mindfulness training lowers preferences for proenvironmental rewards

**Fig. 3:**
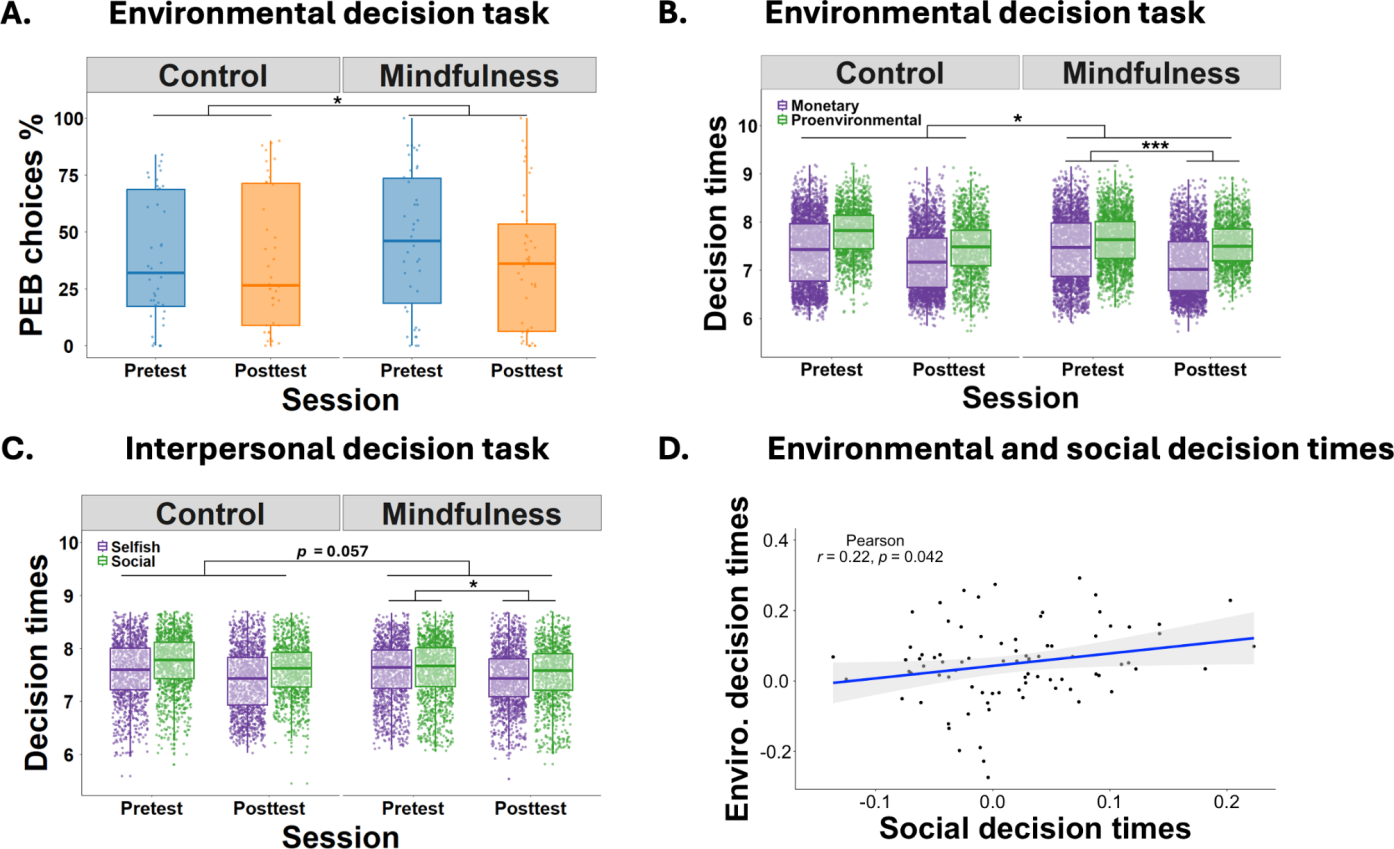
Mindfulness effects on the environmental decision task (**A**,**B**) and interpersonal choice (**C**) task. The mindfulness training, relative to the control training, (**A**) reduced proenvironmental behavior (PEB) choices in the post-test (orange) compared with the pre-test (blue), and (**B**) decreased the decision time for monetary (purple) relative to proenvironmental (green) choices. (**C**) The mindfulness training also decreased the decision time for selfish (purple) relative to social (green) choices, and this effect tended to be stronger in the mindfulness compared with the control group. Lastly, (**D**) the mindfulness effects on decision times in the proenvironmental and interpersonal decision task were positively correlated.

In the environmental decision task, we tested our hypothesis that the mindfulness training increases proenvironmental choices. The interventions differently affected choices in the post-test relative to the pre-test, Group × Session: β = -0.68, *z* = 2.35, *p* = 0.019 (Table S1). To resolve this interaction, we conducted separate GLMMs for each training group: the mindfulness training decreased proenvironmental choices in the post-test compared to the pre-test session, Session: β = -0.79, *z* = 3.92, *p* < 0.01, while the control training did not show significant effects, Session: β = -0.06, *z* = 0.29, *p* = 0.78 (Fig. 3A). Thus, contrary to our hypothesis, the mindfulness intervention reduced rather than increased preferences for proenvironmental outcomes. To explore whether the unexpected direction of this effect could be explained by individual differences in participants’ baseline proenvironmental behavior, baseline mindful state, or gender, we added in separate models the mean percentage of proenvironmental choices from the pre-test, the baseline in MAAS score (MAAS score during pre-test), and gender to the model described above. Importantly, the Group × Session interaction remained significant when controlling for individual differences in baseline proenvironmental behavior, β = -0.66, *z* = 2.16, *p* = 0.03, baseline mindful state, β = -0.67, *z* = 2.35, *p* = 0.02, and gender, β = -0.69, *z* = 2.40, *p* = 0.02, and was not significantly modulated by baseline proenvironmental behavior, β = 0.24, *z* = 0.69, *p* = 0.49, baseline in mindful state, β = -0.36, *z* = 1.26, *p* = 0.21, or gender: β = 0.2, *z* = 0.76, *p* = 0.45. Moreover, we also added these individual differences (baseline proenvironmental behavior, baseline mindful state, and gender) to the models for the mindfulness group. The negative effects of mindfulness training on proenvironmental choices (post-test compared to pre-test session) remained significant when controlling for individual differences in baseline proenvironmental behavior, β = -0.64, *z* = 2.30, *p* = 0.02, baseline mindful state, β = -0.60, *z* = 3.96, *p* < 0.01, and gender, β = -0.78, *z* = 3.9, *p* < 0.01. Choice behavior was not significantly modulated by baseline proenvironmental preferences, β = 0.01, *z* = 0.02, *p* = 0.98, baseline in mindful state, β = 0.06, *z* = 0.20, *p* = 0.84, or gender: β = 0.04, *z* = 0.20, *p* = 0.84. Thus, the unexpected effect of the mindfulness training on proenvironmental choices cannot be explained by individual differences in proenvironmental preferences, mindful state, or gender. Moreover, to better understand the relationship between participants’ proenvironmental behavior and mindfulness state, we correlated these measures for the pre-test session (PEB pre vs. MAAS pre), post-test session (PEB post vs. MAAS post), and the changes between sessions (PEB diff vs. MAAS diff; i.e., post-test minus pre-test scores) (Table 1). None of these correlations was statistically significant, for all *p* > 0.46.

**Table 1:** Spearman correlations between proenvironmental choice behavior (PEB) and MAAS (mindful attention awareness state) scores for pre-test session, post-test session, as well as the difference between both post-test and pre-test measures.

Variables	Correlation coefficient (rho)	*p*-value
PEB pre and MAAS pre	− 0.08	0.46
PEB post and MAAS post	0.11	0.31
PEB diff and MAAS diff	− 0.11	0.34

In addition to binary choices, we also analyzed decision times as a measure of the strength of participants’ preference for proenvironmental over monetary options (with stronger preferences being indicated by faster decision times^61^). The trainings differentially affected post-test relative to pre-test decision times for proenvironmental versus monetary choices, Group × Session × Choice: β = 0.08, *t*(68) = 2.50, *p* = 0.01 (Table S2). Separate GLMMs for each group suggested that the mindfulness training decreased decision times for monetary relative to proenvironmental choices in the post-test relative to the pre-test, Session × Choice: β = 0.09, *t*(32) = 4.66, *p* < 0.001, whereas we observed no significant effects for the control training, β = 0.01, *t*(35) = 0.28, *p* = 0.78 (Fig. 3B). Taken together, the mindfulness training, compared with the control training, increased the preference for monetary relative to proenvironmental outcomes.

**Fig. 4:**
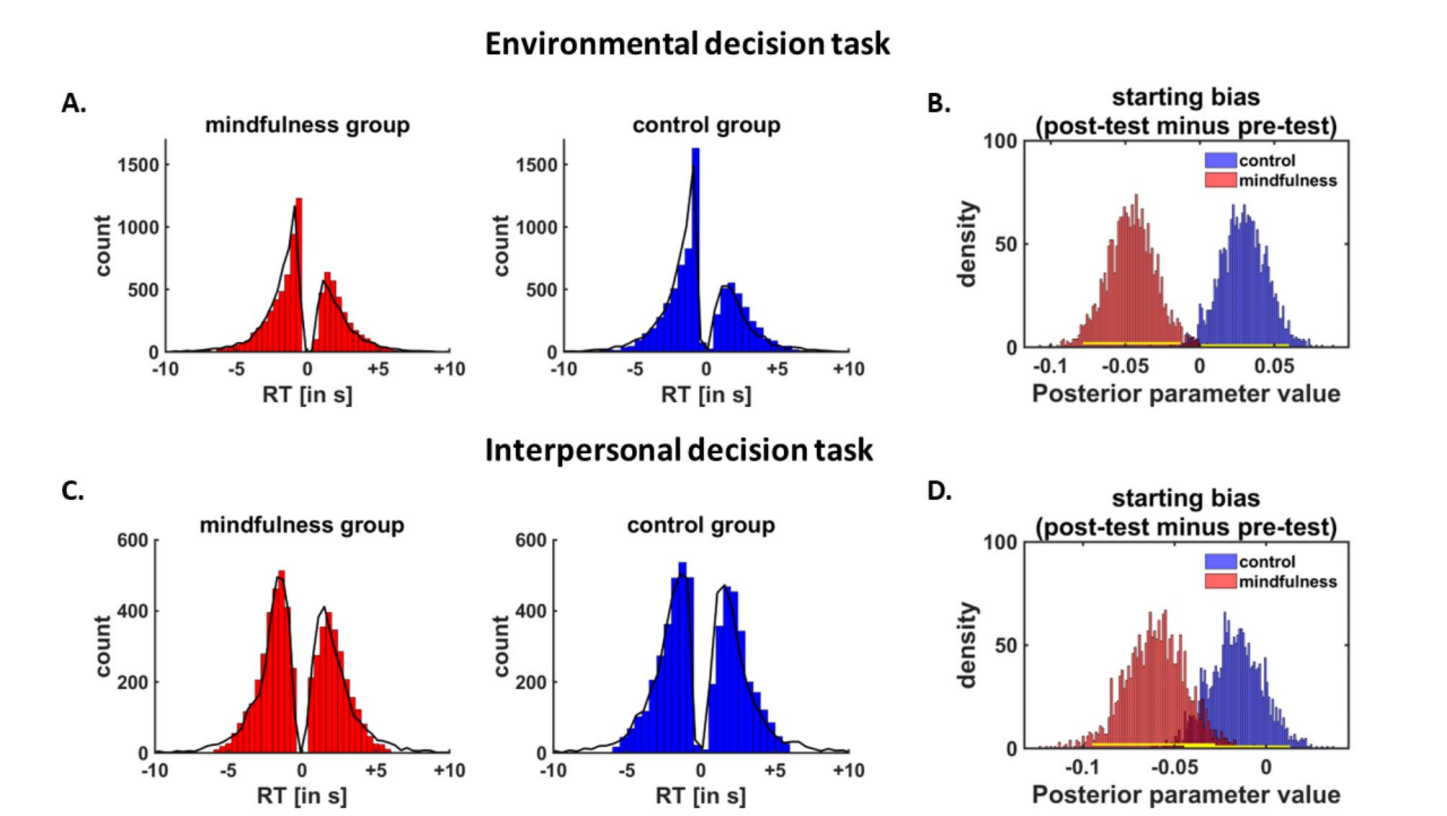
Illustration of the posterior predictive check and drift diffusion model results for the environmental decision task and interpersonal decision task. For the posterior predictive check, simulated reaction times (based on estimated drift diffusion model parameters) were compared with observed decision times in the mindfulness and control group (**A**,**C**). The mindfulness training shifted the starting bias parameter towards monetary choices (lower decision boundary) in the environmental decision task (**B**) and towards selfish choices (lower decision boundary) in the interpersonal decision task (**D**) in the post-test relative to the pre-test.

To corroborate the finding that mindfulness training strengthens the preference for monetary options, we analyzed data in the environmental decision task also with hierarchical Bayesian DDMs. DDMs explain observed choices and decision times via an evidence accumulation process, where individuals accumulate evidence for the options from the starting point ζ until the strength of the accumulated evidence surpasses the decision boundary α. We assumed that the velocity ν of the accumulation process depends on the weighted influences of the rewards and reduction of carbon dioxide emissions on the choice process. Posterior predictive checks comparing simulated decision times (based on estimated DDM parameters) with observed decision times suggested that our model provided a reasonable account of the empirical data (Fig. 4A). As to be expected, both the mindfulness and the control group accumulated evidence faster towards the monetary option the higher participants’ payoff in the monetary option (mindfulness group: HDI_mean_ = -1.12, HDI_95%_ = [-1.44; -0.81]; control group: HDI_mean_ = -1.31, HDI_95%_ = [-1.66; -0.99]) as well as the smaller the difference in carbon dioxide emission reduction between the options (mindfulness group: HDI_mean_ = -0.90, HDI_95%_ = [-1.15; -0.47]; control group: HDI_mean_ = -0.77, HDI_95%_ = [-1.06; -0.47]). When we tested for significant training effects on DDM parameters, we found that the mindfulness intervention significantly shifted the starting point of the accumulation process towards the monetary option in the post-test relative to the pre-test, HDI_mean_ = -0.05, HDI_95%_ = [-0.08; -0.01], and this effect was significantly stronger than in the control group, HDI_mean_ = -0.08, HDI_95%_ = [-0.12; -0.03] (Fig. 4B). In contrast, we observed no significant group differences between the influences of reward, HDI_mean_ = -0.17, HDI_95%_ = [-0.61; 0.29], and of carbon dioxide emission on the drift rate, HDI_mean_ = 0.18, HDI_95%_ = [-0.14; 0.51], as well as on decision boundaries, HDI_mean_ = -0.05, HDI_95%_ = [-0.28; 0.20], and non-decision times, HDI_mean_ = -0.01, HDI_95%_ = [-0.14; 0.11]. Thus, the DDM analysis replicates the finding that the mindfulness training increased the preference for monetary options and provides insights into the subcomponent of the choice process that was altered by the mindfulness intervention.

In contrast to the environmental decision task, there were no significant training effects in the environmental donation task, β = 0.15, *t*(80) = 0.43, *p* = 0.67, or in proenvironmental attitudes measured by the NEP scale^56^, β = -0.003, *t*(80) = 0.06, *p* = 0.96. The latter suggests that mindfulness training did not affect self-reported proenvironmental attitudes despite lowering choice-revealed preferences for proenvironmental outcomes in the environmental decision task.

### Mindfulness training changed social but not future-oriented preferences

The unexpected direction of the impact of the mindfulness training on proenvironmental behavior raises the question as to how the stronger preference for monetary options in the mindfulness group can be explained. Proenvironmental decisions are conceptually linked to prosocial and future-oriented preferences^7,45,62^. Given the unexpected training effects on proenvironmental behavior, we reasoned that (contrary to our original hypothesis) the mindfulness intervention might increase the preference for selfish or immediate options in the interpersonal and intertemporal decision tasks, respectively. Baseline preferences for proenvironmental rewards (pre-test) were significantly correlated with pre-test choices in the interpersonal, ρ = 0.59, *p* < 0.01, but not in the intertemporal decision task, ρ = 0.049, *p* = 0.66. In neither of these tasks, we observed significant training effects (Group × Session interactions) on binary choices, both *z* < 0.07, both *p* > 0.94 (Table S3 and S5), or on hyperbolic discount parameters, all *t* < 0.49, all *p* > 0.62. However, an analysis of decision times in the interpersonal decision task suggested that the trainings tended to have dissociable effects on post-test relative to pre-test decision times for prosocial versus selfish choices, Group × Session × Choice: β = 0.05, *t*(64) = 1.94, *p* = 0.057 (Fig. 3C, Table S4). Separate GLMMs for each group suggested that the mindfulness training increased decision times for prosocial relative to selfish choices in the post-test relative to the pre-test, Session × Choice: β = 0.04, *t*(33) = 2.06, *p* = 0.047, whereas the control training showed no effect, β = -0.01, *t*(32) = 0.56, *p* = 0.58. Thus, in analogy to the findings for the environmental decision task, the mindfulness training tended to increase the preference for selfish rewards in the interpersonal decision task. To identify whether there is a correlation between the mindfulness effects on decision times in the environmental and the interpersonal decision task, we correlated the model parameters capturing the training effects on decision times in these tasks, which revealed a small to moderate correlation, *r* = 0.22, *p* = 0.042 (Fig. 3D). This suggests that stronger training-induced changes in interpersonal decisions were also associated with more pronounced training effects on proenvironmental decisions.

To ensure that our results were not influenced by the number of training sessions completed by participants, we re-ran the model-free analysis (GLMMs) for proenvironmental, prosocial, and future-oriented behavior (both on choices and reaction time models), excluding participants who missed at least two sessions during the 31 days. Seven participants were excluded in total. In the environmental decision task, these control analyses replicated previous training effects on proenvironmental choices (Group × Session: β = -0.75, z = 2.45, *p* = 0.01) and on decision times (Group × Session × Choice: β = 0.07, *t*(64) = 2.07, *p* = 0.04). As for the models including all participants, mindfulness effects on prosocial and intertemporal choices were not significant, both *z* < 0.36, both *p* > 0.72. Moreover, the re-analysis of decision times in the interpersonal decision task showed that, as in the original analysis, the trainings tended to have dissociable effects on post-test relative to pre-test decision times for prosocial versus selfish choices (Group × Session × Choice: β = 0.05, *t*(58) = 1.85, *p* = 0.07). On balance, this provides little evidence for an influence of the number of completed training sessions on the current results, at least in the environmental decision task.

In analogy to the proenvironmental behavior task, we again fitted Bayesian DDMs to the data in the interpersonal decision task and the posterior predictive checks in this task also suggested that our model provided a reasonable account of the empirical data (Fig. 4C). Again, the mindfulness training affected the starting bias parameter (shifting the bias towards the selfish option), HDI_mean_ = − 0.06, HDI_95%_ = [-0.09; − 0.03], and this effect was significantly stronger in the mindfulness compared with the control group, HDI_mean_ = − 0.04, HDI_95%_ = [− 0.09; − 0.00] (Fig. 4D). The starting bias parameter in the baseline pre-test session was significantly correlated between the environmental and the interpersonal decision task, *r* = 0.41, *p* < 0.001, and also the training effects on the starting bias showed a trend-level positive correlation, *r* = 0.19, *p* = 0.08. The mindfulness training, relative to the control training, also significantly increased the decision boundary parameter, HDI_mean_ = 0.25, HDI_95%_ = [0.08; 0.43], suggesting that participants made more cautious decisions after the mindfulness training (i.e., accumulated more evidence before making a choice). No further parameter showed significant training effects in the interpersonal decision task. In the intertemporal decision task, there were no significant differences in DDM parameter estimates between the mindfulness and the control group (all HDI_95%_ included zero). Taken together, the DDM and the model-free analyses provide converging evidence for stronger preferences for monetary and selfish rewards after the mindfulness compared with the control intervention.

To determine whether participants’ education levels influenced our results, we conducted a control analysis by re-running all GLMMs for proenvironmental, intertemporal, and interpersonal decision tasks (binary choices and decision times). We included education as a fixed-effect predictor (0 = both university entrance diploma and vocational school diploma, as these are considered equivalent in the German educational system; 1 = completed university degree). The effects of the mindfulness training remained significant for proenvironmental choices (Group × Session: β = − 0.68, *z* = 2.38, *p* = 0.017), proenvironmental decision times (Group × Session × Choice: β = 0.08, *t*(68) = 2.51, *p* = 0.01), and showed a tendency towards significance for prosocial decision times (Group × Session × Choice: β = 0.05, *t*(64) = 1.95, *p* = 0.056), mirroring the main results. Consistent with our primary findings, we observed no significant mindfulness training effects on binary choices for interpersonal or intertemporal tasks, both z < 0.067 and *p* > 0.94. Therefore, the current findings are robust in controlling for individual differences in education level.

The significant training effects on proenvironmental and interpersonal decisions raise the question as to whether the impact of the mindfulness training on proenvironmental preferences can be statistically explained by the training effects on social preferences. To test for such a mediation effect, we regressed individual parameters capturing the training effect on the starting bias in the proenvironmental decision task on predictors for Group (mindfulness versus control) and individual parameters for the training effect on the starting bias in the interpersonal decision task. While the effect of Group remained significant, *z* = 4.60, *p* < 0.001, bias parameters from the interpersonal decision task did not significantly explain variance in the proenvironmental decision task, *z* = 0.33, *p* = 0.74. Moreover, also the non-significant Sobel test (measuring the influence of the indirect mediation path) provided no evidence for a mediation effect, *p* = 0.74. Thus, our data do not support the assumption that mindfulness training affected proenvironmental preferences by increasing selfishness in interpersonal decisions.

## Discussion

The goal of the present study was to test whether mindfulness enhances proenvironmental preferences via strengthening prosociality or future orientation. Contrary to our original hypotheses, the mindfulness training reduced preferences for proenvironmental outcomes in the environmental decision task (hypothesis 1) and prosocial outcomes in the interpersonal decision task (hypothesis 2) in both model-free and model-based analyses. In the model-free analyses, negative influences of mindfulness on proenvironmental preferences were evidenced by training effects on both choices and decision times, with longer decision times (indicating weaker preferences^61^) for proenvironmental versus monetary options after the mindfulness training. Our hierarchical Bayesian DDMs moreover provided insights into the subcomponent of the decision process underlying these effects: the mindfulness training shifted the starting point of the evidence accumulation process towards monetary options without affecting the evaluation of reward magnitudes or action costs. Note though that we observed no training effects on the environmental donation task, potentially due to the limited number of trials in this task (only four donations compared with the 100 decisions in the proenvironmental decision task).

In our study, which elicited proenvironmental preferences through a decision task with real consequences for both decision-makers and the environment, we observed that a mindfulness intervention might reduce the preference for proenvironmental outcomes. This finding challenges accounts according to which mindfulness should be linked with stronger proenvironmental preferences^7,23^. While this hypothesis was mainly based on correlative evidence^23^, intervention studies observed no direct mindfulness effects on self-report measures of proenvironmental behavior^29^. Interestingly, the mindfulness intervention unexpectedly also enhanced the preference for selfish over prosocial rewards in the interpersonal decision task (hypothesis 2). This finding appears to be at variance with a recent meta-analysis suggesting a positive connection between mindfulness and prosocial behavior^42^.

There are several possible explanations for this discrepancy. One explanation could be a u-shaped relationship between training length and effects on proenvironmental or prosocial behavior, where a medium training length might reduce and longer trainings might enhance proenvironmental or prosocial preferences. However, previous studies provide no evidence for an u-shaped relationship between training length and training effects on proenvironmental or prosocial preference^31,42^.

Second, previous mindfulness studies on prosociality mainly relied on self-report measures^42^ or hypothetical scenarios^63^, contrary to our task where sharing involved real monetary consequences for the participants and the benefitted others. Interestingly, one study reported mindfulness to increase acceptance rates for unfair offers in the ultimatum game^64^, which was interpreted as increased cooperativeness, although accepting unfair offers in the ultimatum game maximizes also a decision maker’s selfish payoff; this, in turn, is consistent with our findings where mindfulness training strengthened the preference for options with larger selfish rewards. Taken together, the meta-analytical evidence for mindfulness effects on prosociality should be interpreted with caution, given the small number of experimental (compared with self-report) measures of prosociality involving real consequences for self and others^42^. This is consistent with another meta-analysis^65^, which previously questioned the beneficial effects of mindfulness interventions on prosocial behavior by showing that the effects of mindfulness trainings varied depending on the type of prosocial behavior studied (i.e., aggression, compassion, empathy, prejudice, or connectedness). Beneficial mindfulness effects were limited to compassion and empathy and were only observed in studies with methodological limitations, such as exclusively using passive controls and the mindfulness teachers being co-authors of the papers.

Third, a recent study suggests that the mindfulness effects depend on the specific content of the training, for instance, completing a module focused on compassion and loving-kindness meditation, resulted mostly in higher compassion, whereas attention improved most after attention training^12^. Our training involved only a few sessions focusing on the relationship of the participant with others, in contrast to trainings in other studies which put more weight on such social aspects of mindfulness^66^. Interestingly, mindfulness trainings focusing on self-centered aspects resulted in less proenvironmental intentions, while mindfulness interventions focusing on social or biospheric contents enhanced proenvironmental intentions^25^. Given that most of our mindfulness exercises focused on self-centered content, a mindfulness training focusing on social or biospheric content might strengthen instead of weaken proenvironmental and prosocial preferences^67,68^. Thus, we recommend future studies to use a less generalized mindfulness training and to increase the number of mindfulness exercises focused on specific social components such as loving-kindness meditations and environmental elements, for instance by adding natural sounds to the mindfulness exercises^69^, or even conducting the mindfulness exercises in nature. Alternatively, studies could incorporate exercises that promote mindful engagement with nature and encourage participants to reflect on their connectedness with it. Moreover, to determine whether the specific content of the training influences the direction of its effects on proenvironmental behavior (or prosocial behavior), we suggest that future studies directly compare trainings with and without natural components (or social components).

Next, individual differences might influence the effects of mindfulness on proenvironmental and prosocial behavior. For instance, it has been suggested that mindfulness effects on social preferences may depend on inter-individual variation in how separate individuals perceive themselves from the others^70^. Therefore, we recommend future studies to identify participants who are more responsive to mindfulness training related to environmental behavior by considering inter-individual differences, such as socioeconomic status, individual stress level, or personality traits^71,72^.

Lastly, based on previous findings that lowering the excitability of the prefrontal cortex as neural substrate of self-control promoted proenvironmental decisions^73^, a further possibility is that the mindfulness training reduced proenvironmental decisions via strengthening self-control processes (though note that the lack of training effects on intertemporal choices seems at variance with this assumption).

To summarize, the influence of mindfulness interventions on proenvironmental and prosocial choices may strongly depend on the training characteristics and the individual susceptibility to training effects^74^. While our findings should not be misinterpreted as evidence that mindfulness generally promotes selfish or non-environmental behavior, they appear consistent with recent accounts according to which contemporary mindfulness training might strengthen self-focused behavior^75^. Moreover, while our findings provide novel insight into the relationship between mindfulness, proenvironmental, and prosocial behavior, several limitations should be acknowledged as they might have influenced our results. First, although pseudorandomization and researcher blindness were implemented, a computer-based randomization method would even further have reduced the risk of potential biases in group assignment. Furthermore, participants who did not complete the training were automatically excluded from the post-test session. This exclusion could introduce bias, as those who missed sessions might systematically differ from those who completed the training, potentially impacting the generalizability of our findings. Lastly, we did not record participants’ socioeconomic status, which might potentially have impacted our findings.

Prosocial and proenvironmental preferences showed a moderate positive correlation during the baseline pre-test session in both model-free and model-based analyses. Additionally, the intervention effects on these preferences exhibited a weak positive correlation in the model-free analysis and tended to covary in the model-based analysis. While this supports the hypothesized link between proenvironmental and prosocial behavior (which so far relied only on self-report questionnaire measures^33^ ), it is important to note that we observed no evidence for a significant mediation effect. In other words, the current data do not allow concluding that the mindfulness training reduced proenvironmental preferences via increasing selfishness. Instead, the significant correlation but lack of a mediation effect speaks in favor of a third variable that was affected by the training and resulted in the observed training effects on proenvironmental and prosocial preferences. Mindfulness training was suggested to reduce habitual behavior^7^, but the current data provide no evidence that proenvironmental and pro-social decisions might represent the habitual responses in the current tasks (as participants chose the proenvironmental and pro-social options in only 42% and 50% of all pre-test decisions, respectively). We also observed no significant training effects on mood or working memory capacity, which were linked to proenvironmental and prosocial choices^7,76,77^. A further potential explanation for the correlated mindfulness effects on proenvironmental and social preferences is that mindfulness reduces feelings of guilt. According to Baumgartner et al. (2021), individuals who reported guilt after having been warned about the limited availability of resources were more likely to minimize resource depletion (proenvironmental decision)^78^. Likewise, another study showed that guilt increased cooperation in a social dilemma game that focused on shared electricity usage at home^79^. The mindfulness effects on both proenvironmental and prosocial preferences in our study might thus be related to reduced feelings of guilt^80^, though this explanation remains speculative given that our study included no measure of guilt. We therefore recommend future studies to measure mindfulness effects on feelings of guilt associated with non-environmental choices. Moreover, connectedness to nature has been proposed as a potential mediator between mindfulness and proenvironmental behavior. Barbaro et al. demonstrated that connectedness to nature indirectly influences the relationship between dispositional mindfulness and self-reported proenvironmental behavior^81^. Supporting this, a recent intervention study showed that connectedness to nature partially explains the effect of mindfulness on proenvironmental behavior^69^. Therefore, we recommend that future studies incorporate measures of connectedness to nature in their study design. While the current data cannot provide a conclusive answer to the question of why the mindfulness intervention reduced proenvironmental and prosocial preferences, they nevertheless provide evidence that mindfulness might not necessarily lead to more proenvironmental behavior, contrary to theoretical assumptions.

Our study revealed no mindfulness effects on future-oriented preferences measured with the intertemporal decision task, which may be unsurprising given the inconsistent mindfulness effects on time preferences in previous studies^43,44,82^. Here too, heterogeneity in administered tasks, specific contents of the mindfulness training, and baseline time preferences (participants chose the larger-later option in 75% of all baseline decisions, leaving little room for training effects to further increase patience) may play a crucial role for determining the influence of a mindfulness intervention on time preferences.

Taken together, we provide evidence that a mindfulness intervention can reduce proenvironmental and prosocial preferences and that the influences of mindfulness on these preferences might be correlated. This challenges existing theories about the positive impact of mindfulness on proenvironmental and prosocial behavior and suggests that the effects of mindfulness on proenvironmental and social behavior might be more complicated than previously assumed based on correlative evidence. The current findings moreover advance our understanding of the psychological mechanisms underlying proenvironmental decision by showing that these appear to be more strongly linked to prosociality than to future orientation. These insights into the psychological determinants of proenvironmental decisions may deepen the understanding of the reasons why humans often fail to act in environmentally friendly ways despite their best intentions and pave the ground for designing interventions for promoting proenvironmental behavior.

## Electronic supplementary material

Below is the link to the electronic supplementary material.

Supplementary Material 1

## Data Availability

The behavioral data supporting the findings of this study and the data analysis code will be available on Open Science Framework under the following link (https://osf.io/3wbs4).
