# Supplementary material for: Mindfulness training reduces the preference for proenvironmental outcomes
Source: Sci Rep. 2024 Nov 27;14:29526. doi: 10.1038/s41598-024-79137-0 (PMC11603186; doi:10.1038/s41598-024-79137-0)
Supplement: Supplementary file 1 — Supplementary Material 1 [file 41598_2024_79137_MOESM1_ESM.docx]

**Supplementary materials**

*Table S1.* GLMM results for pro-environmental choices. Group was coded as 0 for the control training and 1 for the mindfulness training, Session was coded as 0 for the pretest session and 1 for the posttest session.

| Fixed Effects | Beta | z | p |
| --- | --- | --- | --- |
| Intercept | -0.92 | 2.8 | <0.01 |
| Group | 0.50 | 1.10 | 0.27 |
| Session | -0.09 | 0.41 | 0.68 |
| Group × Session | -0.68 | 2.35 | 0.02 |

| Random Effects | Variance | Std. Dev. | Correlation |
| --- | --- | --- | --- |
| Intercept | 4.11 | 2.03 |  |
| Session | 1.45 | 1.20 | -0.04 |

*Table S2.* GLMM results for pro-environmental decision times. Group was coded as 0 for the control training and 1 for the mindfulness training, Session was coded as 0 for the pretest session and 1 for the posttest session.

| Fixed Effect | Beta | df | t | p |
| --- | --- | --- | --- | --- |
| Intercept | 7.62 | 76 | 110.21 | <0.001 |
| Group | 0.003 | 76 | 0.03 | 0.97 |
| Session | -0.22 | 79 | 4.6 | <0.001 |
| Choice | 0.06 | 57 | 1.98 | 0.052 |
| Group × Session | -0.04 | 79 | 0.56 | 0.58 |
| Group × Choice  Session × Choice | -0.03  0.01 | 57  68 | 0.84  0.26 | 0.40  0.79 |
| Group × Session × Choice | 0.08 | 68 | 2.50 | 0.01 |

| Random Effects | Variance | Std. Dev. | Correlation |  |  |
| --- | --- | --- | --- | --- | --- |
| Intercept | 0.19 | 0.43 |  |  |  |
| Session | 0.08 | 0.29 | -0.41 |  |  |
| Choice | 0.03 | 0.17 | -0.57 | 0.03 |  |
| Session × Choice | 0.02 | 0.12 | 0.19 | -0.30 | -0.03 |
| Residual | 0.16 | 0.40 |  |  |  |

*Table S3.* GLMM results for prosocial choices. Group was coded as 0 for the control training and 1 for the mindfulness training, Session was coded as 0 for the pretest session and 1 for the posttest session.

| Fixed Effect | Beta | z | p |
| --- | --- | --- | --- |
| Intercept | 0.04 | 0.16 | 0.87 |
| Group | -0.05 | 0.13 | 0.89 |
| Session | -0.35 | 2.12 | 0.03 |
| Group × Session | -0.02 | 0.07 | 0.95 |

| Random Effects | Variance | Std. Dev. | Correlation |
| --- | --- | --- | --- |
| Intercept | 2.33 | 1.53 |  |
| Session | 0.87 | 0.93 | -0.32 |

*Table S4.* GLMM results for prosocial decisions times. Group was coded as 0 for the control training and 1 for the mindfulness training, Session was coded as 0 for the pretest session and 1 for the posttest session.

| Fixed Effect | Beta | df | t | p |
| --- | --- | --- | --- | --- |
| Intercept | 7.70 | 73 | 135.12 | <0.001 |
| Group | -0.08 | 73 | 1.00 | 0.32 |
| Session | -0.17 | 76 | 4.78 | <0.001 |
| Choice | 0.04 | 53 | 1.91 | 0.06 |
| Group × Session | 0.06 | 77 | 1.18 | 0.24 |
| Group × Choice  Session × Choice | -0.03  -0.01 | 54  63 | 0.81  0.47 | 0.42  0.64 |
| Group × Session × Choice | 0.05 | 64 | 1.94 | 0.057 |

| Random Effects | Variance | Std. Dev. | Correlation |  |  |
| --- | --- | --- | --- | --- | --- |
| Intercept | 0.13 | 0.36 |  |  |  |
| Session | 0.04 | 0.21 | -0.23 |  |  |
| Choice | 0.01 | 0.12 | -0.30 | 0.16 |  |
| Session × Choice | 0.01 | 0.09 | -0.23 | -0.04 | -0.46 |
| Residual | 0.15 | 0.38 |  |  |  |

*Table S5.* GLMM results for intertemporal choices (future-oriented choices). Group was coded as 0 for the control training and 1 for the mindfulness training, Session was coded as 0 for the pretest session and 1 for the posttest session.

| Fixed Effect | Beta | z | p |
| --- | --- | --- | --- |
| Intercept | 1.54 | 7.33 | <0.001 |
| Group | -0.08 | 0.26 | 0.79 |
| Session | -0.10 | 0.60 | 0.55 |
| Group × Session | -0.01 | 0.06 | 0.95 |

| Random Effects | Variance | Std. Dev. | Correlation |
| --- | --- | --- | --- |
| Intercept | 1.56 | 1.25 |  |
| Session | 0.77 | 0.88 | -0.26 |

*Table S6.* GLMM results for intertemporal decision times (future-oriented decision times). Group was coded as 0 for the control training and 1 for the mindfulness training, Session was coded as 0 for the pretest session and 1 for the posttest session.

| Fixed Effect | Beta | df | t | p |
| --- | --- | --- | --- | --- |
| Intercept | 7.50 | 79 | 196.50 | <0.001 |
| Group | 0.01 | 79 | 0.22 | 0.82 |
| Session | -0.06 | 80 | 2.18 | 0.03 |
| Choice | -0.07 | 59 | 4.78 | <0.001 |
| Group × Session | -0.003 | 80 | 0.07 | 0.95 |
| Group × Choice  Session × Choice | 0.01  -0.003 | 59  57 | 0.32  0.21 | 0.75  0.83 |
| Group × Session × Choice | 0.02 | 57 | 1.14 | 0.26 |

| Random Effects | Variance | Std. Dev. | Correlation |  |  |
| --- | --- | --- | --- | --- | --- |
| Intercept | 0.06 | 0.24 |  |  |  |
| Session | 0.03 | 0.16 | -0.46 |  |  |
| Choice | 0.005 | 0.07 | 0.45 | -0.10 |  |
| Session × Choice | 0.003 | 0.05 | -0.33 | 0.08 | -0.39 |
| Residual | 0.11 | 0.33 |  |  |  |
